# Loss of Wwox Perturbs Neuronal Migration and Impairs Early Cortical Development

**DOI:** 10.3389/fnins.2020.00644

**Published:** 2020-06-11

**Authors:** Michele Iacomino, Simona Baldassari, Yuki Tochigi, Katarzyna Kośla, Francesca Buffelli, Annalaura Torella, Mariasavina Severino, Dario Paladini, Luana Mandarà, Antonella Riva, Marcello Scala, Ganna Balagura, Andrea Accogli, Vincenzo Nigro, Carlo Minetti, Ezio Fulcheri, Federico Zara, Andrzej K. Bednarek, Pasquale Striano, Hiroetsu Suzuki, Vincenzo Salpietro

**Affiliations:** ^1^Unit of Medical Genetics, IRCCS Istituto “Giannina Gaslini”, Genoa, Italy; ^2^Laboratory of Veterinary Physiology, School of Veterinary Medicine, Faculty of Veterinary Science, Nippon Veterinary and Life Science University, Musashinoi, Japan; ^3^Department of Molecular Carcinogenesis, Medical University of Łódź, Łódź, Poland; ^4^Fetal and Perinatal Pathology Unit, IRCCS Istituto “Giannina Gaslini”, Genoa, Italy; ^5^Department of Precision Medicine, University of Campania “Luigi Vanvitelli”, Naples, Italy; ^6^Telethon Institute of Genetics and Medicine (TIGEM), Naples, Italy; ^7^Neuroradiology Unit, IRCCS Istituto “Giannina Gaslini”, Genoa, Italy; ^8^Fetal Medicine and Surgery Unit, IRCCS Istituto “Giannina Gaslini”, Genoa, Italy; ^9^Medical Genetics Unit, Maria Paternò Arezzo Hospital, Ragusa, Italy; ^10^Pediatric Neurology and Muscular Diseases Unit, IRCCS Istituto “Giannina Gaslini”, Genoa, Italy; ^11^Department of Neurosciences, Rehabilitation, Ophthalmology, Genetics, Maternal and Child Health (DiNOGMI), University of Genoa, Genoa, Italy; ^12^Department of Surgical Sciences and Integrated Diagnostics (DISC), Pathology Division of Anatomic Pathology, University of Genoa, Genoa, Italy; ^13^Department of Neuromuscular Diseases, Queen Square Institute of Neurology, University College London, London, United Kingdom

**Keywords:** WWOX, WOREE syndrome, neuropathology, animal model, developing brain, cytoskeleton

## Abstract

Mutations in the *WWOX* gene cause a broad range of ultra-rare neurodevelopmental and brain degenerative disorders, associated with a high likelihood of premature death in animal models as well as in humans. The encoded Wwox protein is a WW domain-containing oxidoreductase that participates in crucial biological processes including tumor suppression, cell growth/differentiation and regulation of steroid metabolism, while its role in neural development is less understood. We analyzed the exomes of a family affected with multiple pre- and postnatal anomalies, including cerebellar vermis hypoplasia, severe neurodevelopmental impairment and refractory epilepsy, and identified a segregating homozygous *WWOX* mutation leading to a premature stop codon. Abnormal cerebral cortex development due to a defective architecture of granular and molecular cell layers was found in the developing brain of a *WWOX*-deficient human fetus from this family. A similar disorganization of cortical layers was identified in *lde/lde* rats (carrying a homozygous truncating mutation which disrupts the active Wwox C-terminal domain) investigated at perinatal stages. Transcriptomic analyses of Wwox-depleted human neural progenitor cells showed an impaired expression of a number of neuronal migration-related genes encoding for tubulins, kinesins and associated proteins. These findings indicate that loss of Wwox may affect different cytoskeleton components and alter prenatal cortical development, highlighting a regulatory role of the *WWOX* gene in migrating neurons across different species.

## Introduction

*WWOX* (MIM 605131) encodes a WW domain-containing oxidoreductase known to be involved in several cellular and biological processes, including tumor suppression, cell growth and differentiation, regulation of steroid metabolism, induction of apoptosis and central nervous system (CNS) development (Bednarek et al., 2001; Aldaz et al., 2014; Iatan et al., 2014; Tanna and Aqeilan, 2018). Biallelic pathogenic *WWOX* variants have been implicated in W**WO**X-**r**elated **e**pileptic **e**ncephalopathy (WOREE syndrome; MIM 616211), a very rare and severe neurological disorder characterized by absence of language development and acquisition of walking, drug-resistant seizures, ophthalmological involvement and a high likelihood of premature death (Tabarki et al., 2015a, b; Johannsen et al., 2018; Piard et al., 2018). Consistent with the human disease phenotypes, Wwox knockout (KO) mice exhibit ataxic gait and epilepsy since birth and die prematurely (Mallaret et al., 2014). **L**ethal **d**warfism with **e**pilepsy (*lde/lde*) rats, carrying a spontaneous homozygous frameshift mutation in the gene, show a high incidence of ataxia, audiogenic seizures, dwarfism and risk of premature death (Suzuki et al., 2009), and display pathological features of abnormal myelination and a marked reduction in astrocytes and microglia (Tochigi et al., 2019). To date, however, no study has assessed the impact of pathogenic *WWOX* variants on the development of cerebral cortex, and the neuropathological features of WOREE syndrome have not been investigated before. As a result, disease mechanisms implicating loss of Wwox in CNS development and diseases have not been fully understood yet. In this study, we report a WOREE syndrome family with a 4-year-old male proband presenting wide congenital anomalies, postnatal microcephaly and developmental epileptic encephalopathy, and a male fetus diagnosed by prenatal imaging with cerebellar vermis hypoplasia and terminated at 21th gestational week. A homozygous segregating *WWOX* c.790C > T mutation, predicted to introduce a premature stop codon at protein position 264, was found by analyzing the exomes of the family. Histopathological studies of the fetal brain revealed an abnormal formation of the developing cerebral cortex and similarly, altered cortical layers were identified in the developing brains of the *lde/lde* rats. Analysis of transcriptome of human neural progenitor cells (hNPC) and neurons with silenced Wwox revealed a reduced expression of different cytoskeleton-related genes. Our findings expand pre- and postnatal features associated with pathogenic *WWOX* variants and indicate that Wwox deficiency may affect neuronal migration and impair cerebral cortication, ultimately contributing to WOREE-related clinical and molecular phenotypes.

## Materials and Methods

Patient data were reviewed for the medical history, the genetic and radiological investigations. Whole exome sequencing (WES) was performed after written informed consent was obtained from parents, according to “Giannina Gaslini” Children’s Hospital Institution review board.

### Genetic Studies

Genomic DNA was isolated from 1 ml of peripheral blood using QIAamp DNA Blood Midi kit (Qiagen). Genomic DNA was enriched with SureSelect Clinical research exome 54Mb (Agilent Technologies). Whole exome sequencing (WES) runs were performed on the HiSeq1000 platform (Illumina Inc., San Diego, CA, United States) as 100-base paired-end runs, using a standard Illumina pipeline as previously described (Salpietro et al., 2019). Paired-end reads were mapped to the reference human genome sequence (GRch37/hg19) using CLC Bio Genomics Workbench 7.5.1 software (CLC Bio, Aarhus, Denmark), after removal of duplicates. Single-nucleotide polymorphisms (SNPs) and short deletion or insertion (indels) variants were called by CLC Bio Workbench software using the specific variant calling plugin and dbSNP147 and gnomAD databases. The variants were filtered for in-house variants controls. In accordance with the pedigree and phenotype, our filtering strategy prioritized rare (<1% in public databases, including 1000 Genomes project and gnomAD) biallelic and X-linked hemizygous coding variants and/or variants located in genes previously implicated in microcephaly and developmental epileptic encephalopathies. In order to investigate the presence of homozygosity regions, we performed homozygosity mapping analyzing the proband variant call format (VCF) WES data on HomozygosityMapper^1^. Validation, parental origin of the resulting variants and family segregation studies were performed by Sanger sequencing.

### Neuropathological Studies

After expulsion of the *WWOX*-deficient male fetus at the 21th gestational week, his whole body was fixed in 10% buffered formalin approximately 6 h after termination. Autopsy was performed 8 weeks later. During autopsy, a sample of brain tissue (parietal coronal section) was taken, after the brain hemispheres had been exposed by means of cutting the scissurae of the parietal and frontal bones. We obtained samples of brain tissue from a control male fetus at the 21th gestational week by using the same method and fixed them 4.5 h after death in 10% buffered formalin for 2 weeks. For microscopy, tissue blocks were fixed in formalin and embedded in paraffin in accordance with standard procedures (Jansen et al., 2016). Sections (7 μ) were deparaffinized and hydrated in decreasing ethanol concentrations. Haematoxylin and eosin (H&E; Ventana HE 600 system) was performed in order to obtain a histo-architectural overview. Periodic Acid Schiff (PAS; Ventana Staining Kit 860-014) staining was performed by means of Benchmark special stains (Roche), in order to document the fine structure of the glial trajectories (Mora et al., 2007). Two further immunohistochemical reactions using CD31 (mouse monoclonal antibody JC70 Cell Marque - Roche) and GFAP (Glial Fibrillary Acidic Protein EP672Y rabbit monoclonal antibody 760-4345 Roche) were performed to investigate the superficial vascular network and processes of radial glial cells (Bär, 1980; Arisio et al., 2002; Jansen et al., 2016).

### *lde/lde* Rat Experiments

Inbred Lethal Dwarfism with Epilepsy rat strain carrying loss-of-function mutation on Wwox gene has been maintained in a clean conventional animal room under a controlled light-dark cycle (14:10 h), with a certified diet (CR-LPF; Oriental Yeast Co., Ltd., Tokyo, Japan) and water *ad libitum* (Suzuki et al., 2007). All animal experiments and animal care were approved by Animal Care and Use Committee of Nippon Veterinary and Life Science University (Protocol #2019K-28, 31 March 2019), and were conducted in accordance with the Guidelines of the Animal Care and Use Committee of Nippon Veterinary and Life Science University. Pregnant rats obtained from mating between *+/lde* rats were intraperitoneally injected with 5-bromo-2′-deoxyuridine (BrdU, 100 mg/kg of body weight) (Sigma-Aldrich, St. Louis, MO, United States) at embryonic day (E) 16.5 to evaluate the migration of late-born neuron (Hatanaka et al., 2004; Fan et al., 2008). Resulted pups (both sexes) were dissected under the isoflurane anesthesia at postnatal day 1 (P1). Before removing the brain, whole body was perfused with phosphate-buffered saline (PBS) containing 10 unit/mL heparin followed by 4% buffered paraformaldehyde. After 48 h fixation with paraformaldehyde, isolated brains were embedded in O.T.C compound according to general protocol as previously described (Tochigi et al., 2017). Twenty-μm-thick sequential sagittal cryosections were prepared from 1 to 2 mm lateral region to the midline. Almost corresponding sections were used for comparison. Nissl staining were performed for histological examination. Air-dried cryosections were microwaved with 10 mM sodium citrate (pH 6.0) for 1 min for antigen retrieval. Anti-Satb2 (1:500, clone SATBA4B10, Abcam plc, Cambridge, United Kingdom) and anti-Tbr1 (1:5000, ab31940, Abcam plc) were used to detect layer specific-neuron subtypes (Hevner et al., 2001; Britanova et al., 2005). The positive signals of BrdU were visualized by denaturing with HCl treatment and following immunohistochemical procedure previously described (Tochigi et al., 2017). All histological images were taken on a Biozero BZ-X710 fluorescence microscope using software BZ-X analyzer Ver. 1.3.1.1 (Keyence, Tokyo, Japan). To evaluate the distribution of Satb2, Tbr1, and BrdU-positive cells, cerebral wall was divided evenly into 8 areas from below the marginal zone (MZ) to the intermediate zone (IZ) or 10 areas from below the MZ to the ventricular zone (VZ). The number of positive cells and area (mm^2^) that contains these cells were measured using analytical software Image J (Ver. 1.46r, NIH, Bethesda, MD, United States) (Tochigi et al., 2019). Data from each 3 *+/+* and *lde/lde* rats genotyped by PCR were used for statistical analysis.

### Transcriptomic Analyses of hNPC and Neurons With Silenced Wwox

To analyze the transcriptomic changes caused by *WWOX* depletion in human neuronal progenitor cells (hNPC), and their putative connection to observed disease phenotypes, we employed the data (deposited in GEO Database with accession number GSE126075), obtained in the experiment of *in vitro WWOX* silencing in H9-derived hNPC as previously described by some of us (Kośla et al., 2019). *WWOX* was silenced by a shRNA lentiviral delivery system and the transcriptome was analyzed by CAGE (cap analysis of gene expression) method. Bioinformatic analysis was performed using transcriptomic data from hNPC with silenced *WWOX* before and after differentiation to neurons in relation to control cells with unaltered *WWOX* level. The raw data were processed and analyzed to investigate expression of genes associated with neural differentiation and migration, using a selection from Molecular Signature Database of Broad Institute^2^. From the generated genomic datasets, genes with a fold change >2 were chosen for further investigation using a signaling pathway analysis. The Gene Set Enrichment Analysis (GSEA) allowed for indication of leading-edge genes contribution to neural differentiation and migration (*t*-test with a weighted scoring scheme and a permutation type regarding phenotype, significance threshold set as FDR < 0.25). Global gene expression analysis was performed using NOISeq software with NOISeq-sim algorithm, as previously described (Tarazona et al., 2015) Volcano plots show the differential gene expression profiles of control neural progenitors versus *WWOX*-depleted neural progenitors and control neurons versus *WWOX*-depleted neurons. P significance was calculated according to NOISeq-sim algorithm. Neural crest specific genes discussed in the manuscript are marked in volcano plots. Immunostaining of *WWOX*-deficient brain was also performed (Supplementary Notes) to confirm hints from transcriptomic studies.

## Results

### Antenatal and Postnatal Features of WOREE Syndrome Associated to Homozygous p.Arg264Ter Variant

**FIGURE 1:**
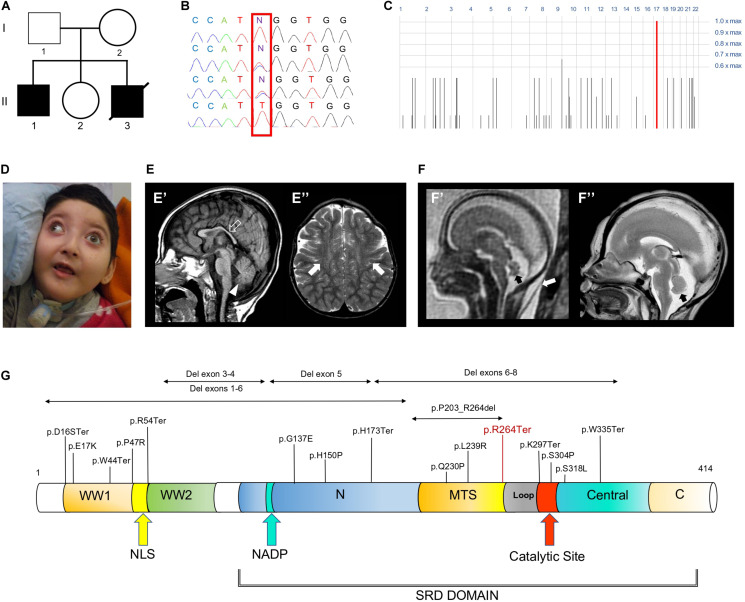
Family tree, genetic studies, WOREE-associated clinico-radiological features, Wwox protein and WOREE-associated mutations. **(A)** Pedigree from Family. **(B)** Electropherograms of carrier parents and index case (II.1) with the c.790C > T homozygous variant introducing a p.Arg264Ter stop codon. **(C)** Homozygosity mapping reveal a homozygous bloc in the WWOX gene 16q23.2 region. **(D)** Distinctive craniofacial features in WOREE syndrome due to homozygous p.Arg264Ter variant, including round hypotonic face and short neck. **(E)** Brain MRI of Patient II.1 performed at 2.4 years of age. **(E’)** Sagittal T1-weighted image reveals hypoplasia of the corpus callosum (empty arrow) and inferior cerebellar vermis (arrowhead). **(E”)** Axial T2-weighted images demonstrates mild atrophy of the frontal lobes with associated bilateral white matter hyperintensity (arrows). **(F)** Fetal MRI **(F’)** and high-resolution post-mortem MRI **(F”)** studies of Patient II.3 performed at 21 gestational weeks demonstrate mild hypoplasia of the cerebellar vermis (black arrows). Note the slightly increased thickness of nuchal subcutaneous tissues on fetal MRI (white arrow). The laminar organization of the cerebral hemispheres and cortical gyration are appropriate for the gestational age (not shown). **(G)** Wwox protein structure and domains and WOREE-associated mutations identified so far.

Patient II.1 was born at 38 weeks after an uneventful pregnancy as the first child to Sicilian non consanguineous healthy parents. There was no history of neurological and/or genetic diseases in the family. At birth growth parameters were within normal limits. Bilateral clubfoot and cryptorchidism were noticed since the first day of life. During the neonatal period, poor spontaneous movements and joint contractures were also observed. At the age of 45 days, epilepsy started with generalized tonic-clonic seizures occurring daily and lately evolving into multidrug-resistant tonic and myoclonic seizures. At the age of 3 years, a percutaneous endoscopic gastrostomy (PEG) tube was placed due to feeding difficulties and failure to thrive. At the time of this report, the boy is 4 years old and he has not acquired any developmental milestones. His occipitofrontal circumference (OFC) is 43 cm (<3.8 SD). He shows some distinctive craniofacial features, including a round hypotonic face and short neck (Figure 1D). He shows no response to visual stimuli and fundoscopy examination revealed bilateral pale optic disks, suggestive of optic atrophy. Neurological examination showed severe truncal hypotonia, arthrogryposis, spastic tetraparesis associated with some dystonic movements, brisk deep tendon reflexes and clonus. During early infancy, extensive diagnostic and neurometabolic workup yielded normal results and cerebrospinal fluid analysis was unremarkable. Electroencephalogram (EEG) studies identified a burst suppression-like pattern in repeated evaluations. Brain magnetic resonance imaging (MRI) performed at the age of 9 months and 2.4 years demonstrated reduction of brain volume, T2 signal alterations of the parietal periventricular and frontal white matter, and hypoplasia of the cerebellar vermis and corpus callosum (Figure 1E). Patient II.3 was a fetus terminated at 21th gestational week, after the third pregnancy of this Sicilian couple (Figure 1A). During pregnancy, Tesla-3D ultrasound scan revealed increased diastolic blood pressure (>90° percentile), growth parameters were compatible with gestational age. Nuchal lucency was normal. Fetal brain MRI documented mild cerebellar vermis hypoplasia (Figure 1F), that was confirmed on high resolution post-mortem MRI performed after the fetus was terminated at 21th week of gestation.

### Identification of the Homozygous p.Arg264Ter Variant

Whole-exome sequencing (WES) of the index case (II-1, Figure 1A) generated a total of 71,369,726 unique reads, with an average on target depth over 150, and >95% of the target bases covered at least 10X. The homozygous c.790C > T; p.Arg264Ter variant in WWOX (NM_016373) was found within a single block of homozygosity consisting in a 1.58 Mb region on chromosome 16 (chr16: 78387189-79966223), as shown by homozygosity mapping analysis (Figure 1C). No other plausible rare variants were identified in the WES data and the c.790C > T WWOX mutation emerged as the explanation for the disease pathophysiology, being fully consistent with the phenotype of WOREE syndrome and segregating within the family (Figure 1B). The p.Arg264Ter variant is predicted highly deleterious (CADD score = 55) and was not observed in approximately 6,100 individuals of European and African American ancestry (NHLBI Exome Sequencing Project) and was reported as pathogenic in three individuals on ClinVar (accession number: VCV000241105). The p.Arg264Ter homozygous variant was confirmed to segregate with the disease in the family (Figures 1A,B). Prenatal testing by Sanger sequencing of individual II.3 identified the p.Arg264Ter variant in the homozygous state, leading to termination of the fetus at 21th week of gestation.

### Defective Architecture of Cortical Layers in *WWOX*-Deficient Human Developing Brain

**FIGURE 2:**
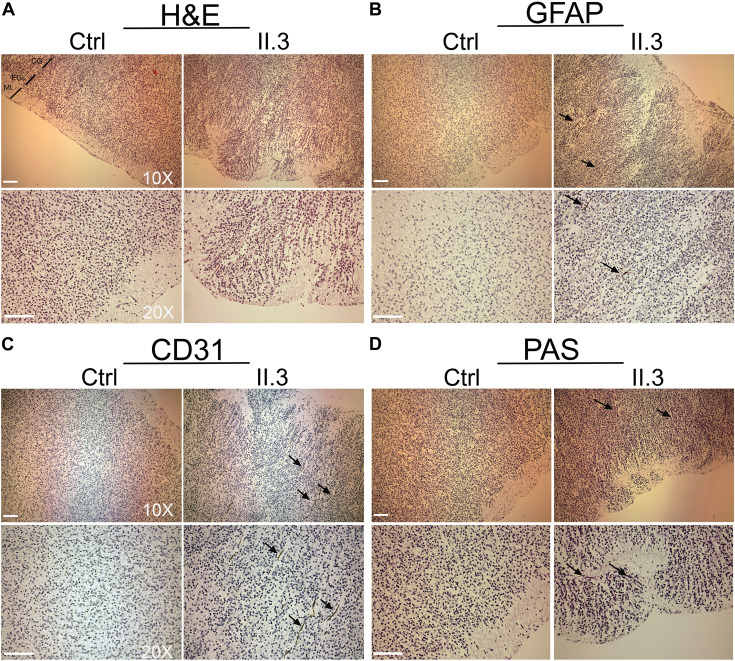
Histopathological studies of *WWOX*-deficient human brain. **(A)** H&E staining shows an incorrect migration of the external granular layer within the molecular layer in patient II.3 (right panel) compared to a normal fetus (left panel) at the same gestational age. Images at 10X and 20X magnification (molecular layer: ML, external granular layer: EGL, cortical gray: CG). Scale bar: 50 μm. **(B)** Histological staining with GFAP 10× (upper panel) and 20× (lower panel) in which a disorganization of the irregularly distributed trajectories is observed (black arrows). Scale bars: 50 μm. **(C)** Histological staining with CD-31 at 10× (upper panel) and 20× (lower panel), in which thinning of the vessels is observed in the context of the external granular layer (black arrows). **(D)** Histological staining with Shift Reactive Periodic Acid (PAS) at 10× (upper panel) and 20× (lower panel) show that the vascular structures of the cortex are irregularly distributed and irregularly branched (black arrows). Scale bars 50 μm.

Apart of cerebellar vermis hypoplasia, there were no anatomical defects or malformations detected at the macroscopic evaluation of the brain of the fetus affected with WOREE syndrome (Supplementary Figures S1A,B). In the histological HE staining of *WWOX*-deficient developing human brain we observed anomalous migration of the external granular layer within the molecular layer and also the latter appeared as not homogeneous (Nishimura, 1983; Figure 2A; Individual II.3). A parietal section of the cerebral cortex of three fetuses of the same gestational period (sampled as above) showed correct cortical development (Figure 2A; Ctrl). Disorganization of irregularly distributed glial trajectories was also observed with GFAP staining (black arrows; Figure 2B, Individual II.3). In the cerebral cortex, a thin vascular network (CD 31-positive) consisting of abnormally thin vessels within the areas below the molecular layer (with marked reduction of these in the context of the external granular layer) was also evident (black arrows; Figure 2C, Individual II.3). Incorrect migration of the external granular layer and inhomogeneous molecular layer were also evident using the PAS staining, which highlighted the texture of the trajectories below the molecular layer of the cortex (black arrows; Figure 2D, Individual II.3).

### Abnormal Neural Migration and Cortical Layer Formation in *lde/lde* Rats

**FIGURE 3:**
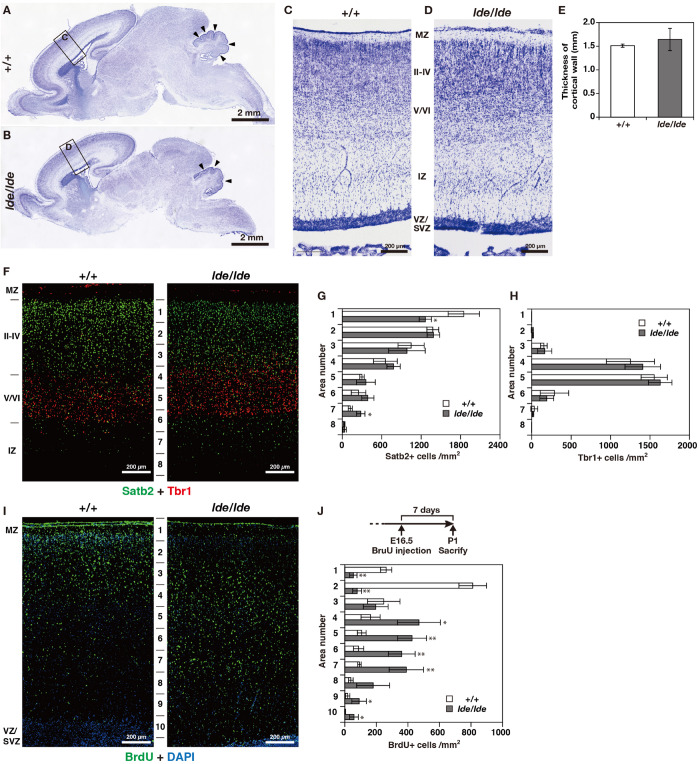
Cortical layer formation and neural migration in *lde/lde* rat brain compared to normal one. **(A–D)** Sagittal brain sections at P1 rats were subjected to Nissl staining. Squares as labeled C and D in panels **(A,B)** indicated identical areas as shown in panels **(C,D)**, respectively. Arrowheads indicate delayed foliation in *lde/lde* cerebellum. **(E)** The thicknesses of cerebral wall (MZ to VZ/SVZ) is shown as mean ± S.D. from three independent experiments. Student’s *t*-test is used to determine statistical significance (^∗^*p* < 0.05). **(F)** Sections are co-immunostained with anti-Satb2 (green) anti-Tbr1 (red). **(I)** Distribution of migrating neuronal cells labeled with BrdU (green) in cerebral cortex. Nuclei are counterstained with DAPI (blue). Representative images from three independent experiments are shown. The cell densities of Satb2 **(G)**, Tbr1 **(H)**, and BrdU-positive cells **(J)** in each area are plotted as histograms. Values are shown as the mean ± SD. The asterisk indicates a significant difference between *+/+* and *lde/lde* in the same area by Student’s *t*-test (^∗^*p* < 0.05, ^∗∗^*p* < 0.01). Experimental scheme to label migrating cells by BrdU is illustrated in panel **(J)**. MZ, marginal zone; IZ, intermediate zone; VZ, ventricular zone: SVZ, subventricular zone.

There were no obvious anatomical defects or significant difference in weight and three-dimensional lengths of brain between *+/+* and *lde/lde* rats at P1 (data not shown). At low magnification of Nissl staining in sagittal brain section, there was no significant difference between +/+ and *lde/lde* in the thickness of cortical wall (from MZ to VZ/SVZ) (Figures 3A–E). In addition, the development of cerebellum was delayed in *lde/lde* as shown by reduced number of foliation (Figures 3A,B, arrowheads). Two developing cortical layers (II-IV and V/VI) were recognized in the cortical plate of +/+, whereas boundary between II-IV and V/VI was unclear in *lde/lde* cortical plate due to irregular cell distribution (Figures 3C,D). To confirm the defects in *lde/lde* cerebral wall, we performed immunohistochemistry with Satb2, a layer II-IV specific marker (Britanova et al., 2005), and Tbr1, a layer V/VI specific marker (Hevner et al., 2001). Two developing cortical layers (II-IV and V/VI) were clearly recognized as positive signals against Satb2 and Tbr1. There was no significant difference between +/+ and *lde/lde* in the distribution of Tbr1- positive cells (Figures 3F,H), whereas the density of Satb2-positive cells was significantly decreased in the surface layer of *lde/lde* cortical plate (Figures 3F,G, area number 1 corresponding to cortical layer II). This defect was accompanied by increase in Satb2-positive cells of the bottom layer in *lde/lde* cerebral wall (Figures 3F,G, area number 7 corresponding to intermediate zone (IZ). Considerable number of Satb2-positive cells and Tbr1-positive cells were mixed in unclear boundary between II-IV and V/VI as shown in Nissl staining of l*de/lde* cortical plate (Figure 3F). Since these results clearly indicated that migration of late-born neuron was impaired in *lde/lde* cortical plate, BrdU was injected fetus at E16.5 when the late-born neuron actively proliferate (Hatanaka et al., 2004; Fan et al., 2008). When BrdU-incorporated cells were traced to P1, we found altered distribution of BrdU-positive cells in *lde/lde* cerebral walls, revealing delayed migration of late-born neurons (Figures 3I,J). These results indicate that Wwox deficiency impairs prenatal neuronal migration which is required for normal cortical layer formation at birth in rats.

### Wwox-Depleted Neurons Show Reduced Expression of Different Neural Migration-Related Genes

**FIGURE 4:**
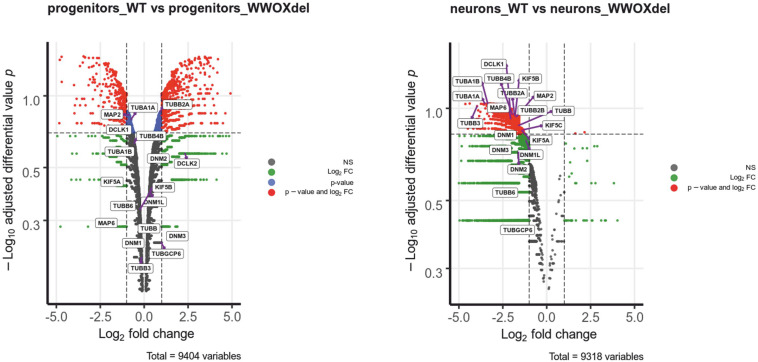
Wwox-depleted neurons show reduced expression of different neural migration-related genes. The differential gene expression profiles in control hNPC vs. WWOX-depleted hNPC and control neurons vs. WWOX-depleted neurons represented in Volcano Plot. P – significance was calculated according to NOISeq-sim algorithm.

Transcriptomic analysis of *WWOX*-silenced human neural progenitors was performed before and after differentiation into mature neurons and focused on genes whose expression was significantly altered after *WWOX* depletion (Log_2_FC < >1). Within this dataset, we identified 20 neural migration-related genes of microtubule architecture and function with the high fold change (>2) of expression (Supplementary Table S1 and Supplementary Figure S2). The relative rank of the genes is presented in volcano plots (Figure 4). Among these, we found tubulin *TUBA1A* gene expression lowered more than 10 times in Wwox-depleted neurons compared to control neurons. Immunostaining in the *WWOX*-deficient human brain revealed a slight reduction of tubulin-1-alpha protein, although not significant (Supplementary Figure S3). Other tubulin-related genes which expression was found to be dramatically reduced include *TUBB3* (>15-fold reduced) and *TUBB2B* (>3-fold reduced expression). Mutations of all three above genes are implicated in a wide array of different human neuronal migration disorders associated with impaired heterodimerization of microtubules. In addition, we found three kinesin-1 heavy chain genes (*KIF5A*, *KIF5B*, and *KIF5C*) showing a 2–3-fold reduced gene expression in Wwox-depleted neurons compared to normal neurons. Also, the *MAP2* gene (encoding a protein involved in stabilizing microtubules by crosslinking each over and with intermediate filaments) expression was found to be 3-fold reduced in Wwox-depleted neurons.

## Discussion

The *WWOX* gene is an important transcriptional regulator, known to modulate the activity of a number of transcriptional factors and molecular pathways (including Jun, AP2gamma, NFkappaB, and ErbB4) and implicated in tumor suppressor activity, steroid-hormone metabolism regulation and brain development (Aldaz et al., 2014; Iatan et al., 2014). The gene encodes Wwox, an oxidoreductase that contains two distinct domains, (a) an amino-terminus showing high sequence conservation to the WW domain family of proteins and displaying two WW domains, and (b) a central 283 amino acids domain homologous to the Short-Chain Dehydrogenase/Reductase (SDR) superfamily (Figure 1G). Wwox shows highest expression in thyroid, testis ovary, brain and cerebellum (Chen et al., 2004; Nunez et al., 2006; Chang et al., 2007). *WWOX* pathogenic biallelic variants are implicated in a spectrum of neurological phenotypes, ranging between autosomal recessive spinocerebellar ataxia type 12 (SCAR12; MIM 614322) to the most severe WOREE syndrome (Gribaa et al., 2007; Abdel-Salam et al., 2014; Mignot et al., 2015). The few SCAR12 individuals described so far harbor two homozygous hotspot missense variants (Pro47Thr and Gly372Arg; NM_016373.4). According to functional studies, these variants cause only partial loss-of-function due to conformational changes that alters Wwox WW1/SDR domains ability to interact with other proteins (Mallaret et al., 2014). WOREE individuals exhibiting absent developmental milestones and profound neurological impairment usually harbor more severe WWOX recessive variants, including nonsense and frameshift variants (Mignot et al., 2015), deletions often involving multiple exons of the gene (Piard et al., 2018), and non-synonymous variants sometimes resulting in full knockout of the protein via impaired *WWOX* translation (Johannsen et al., 2018). In our family, we identified a homozygous p.Arg264Ter variant, resulting in a loss of normal Wwox function either through protein truncation (and disruption of the active C-terminal short-chain dehydrogenase/reductase SDR domain), or nonsense-mediated mRNA decay. Clinical phenotype of the index case (II.1, Figure 1A) was consistent with other individuals reported in the literature and included postnatal microcephaly, absent developmental milestones, visual impairment, tetraparesis, dystonic movements and drug-resistant polymorphic seizures. Moreover, mild cerebral atrophy, white matter signal alterations, and hypoplasia of the cerebellar vermis and corpus callosum were also noted. Interestingly, prenatal features such as reduced fetal movements or congenital clubfoot (occurred in individual II.1) have been occasionally described as part of the WOREE syndrome spectrum (Abdel-Salam et al., 2014; Ben-Salem et al., 2015). A large study on 20 WOREE individuals suggests that antenatal signs, including increased nuchal translucency and hands or feet anomalies, may occur in up to 25% of cases (Piard et al., 2018). Our index case (II.1) was also born with cryptorchidism and, to the best of our knowledge, this feature was not described before as part of the WOREE spectrum. However, testicular atrophy, Leydig cell dysfunction and heterogeneous gonadal abnormalities are usually observed in animal models of Wwox deficiency (Ludes-Meyers et al., 2007; Suzuki et al., 2007). These observations can be explained by the important role of the protein in gonadal development, being its SRD enzymatic domain likely involved in the regulation of sex-steroid metabolism in testis and ovaries (Nunez et al., 2006; Aqeilan et al., 2007, 2009). Notably, WOREE-associated prenatal brain anomalies have been described in 2015 in one fetus carrying a 0.6 homozygous 16q23.1 deletion encompassing *WWOX* (Valduga et al., 2015). In this case, autopsy identified thin corpus callosum and polymicrogyria, but confirmatory brain histopathology studies were not performed. The observation of prenatal brain malformations in WOREE syndrome is consistent with animal studies showing abundant *WWOX* expression in the CNS of mouse embryos, suggesting an important role of the gene in CNS development (Chen et al., 2004). Despite the above studies, disease mechanisms by which loss of Wwox leads to WOREE-associated CNS alterations have not been fully understood yet. Different hypotheses have been proposed, including abnormal tau phosphorylation, mitochondrial dysfunction, altered cell differentiation, and neuronal apoptosis (Ramos and Aldaz, 2006; Aldaz et al., 2014; Liu et al., 2018). To investigate the impact of Wwox deficiency on human brain developmental processes, we performed brain histopathological analyses of the 21th week aborted fetus (II.3) with WOREE syndrome and identified abnormal architecture of the developing cerebral cortex. Development of the cerebral cortex is a very dynamic and highly organized process, involving a series of complex morphogenetic events, which can be impaired in different neurodevelopmental disorders (Fernández-Marmiesse et al., 2019; Seto and Eiraku, 2019; Magdalon et al., 2020). Following division of progenitor cells in the ventricular zone, neurons undergo several physiological changes and migrate outward toward the cortical plate, where they differentiate and integrate into functional neural circuits (Romero et al., 2018). These events are tightly regulated by complex intra- and extra- cellular molecular pathways, including *Notch*, *Wnt*, and *Hedgehog* signaling (Kuzniecky, 2015; Jiang and Nardelli, 2016; Parrini et al., 2016). Importantly, migration of neurons to their final destination sites also requires changes in cell polarity and motility. A pivotal role in modulating these processes is played by cytoskeleton components such as actin filaments, microtubules, and motor proteins (including dyneins and kinesins) which facilitate migrating neurons directional movements (Budday et al., 2015; Jiang and Nardelli, 2016). In human brain fetuses, migration processes begin between the 7th and 8th week of gestation, when neuroblasts migrate along radial extensions of glial cells, to detach when they reach the proper layer of the developing cerebral cortex (Mora et al., 2007). In the present study, HE staining of the *WWOX*-deficient brain revealed abnormal arrangement of cell layers with defective migration of the cerebral external granular layer into the molecular layer, that appeared not homogeneous. In addition, cerebral cortex PAS-stain revealed irregular migration trajectories of glial cells, known to be important in the guidance of migrating neurons. Interestingly, it has been previously showed by some of us (KK, AKB) that Wwox silencing in neural progenitor cells may alter the expression of a large number of genes (e.g., *DCLK*, *NEFM*, and *NEFL*) involved in neurofilament assembly, cytoskeleton organization and chromatin remodeling (Kośla et al., 2019). In the present study, we performed detailed analysis of gene expression of Wwox-depleted neural progenitors and differentiated post-mitotic neurons. This showed a significantly reduced expression of several tubulins, microtubule- associated proteins and microtubule motor kinesins (Figure 4 and Supplementary Table S1). Mutations in genes encoding tubulins may affect neural cell differentiation and neuron migration, resulting in a wide array of brain developmental defects, including lissencephaly-pachygyria and polymicrogyria (Kumar et al., 2010; Poirier et al., 2013; Belvindrah et al., 2017; Juric-Sekhar and Hevner, 2019). These results suggest that abnormal neuronal migration and defective cerebral cortex development can occur as a result of Wwox deficiency. Previously, the clinical features of the *lde/lde* rats, characterized by dwarfism, postnatal lethality, epilepsy and ataxic gait, were related to a spontaneous homozygous 13-bp deletion within the exon 9 of *WWOX*, resulting in a premature truncation of the Wwox C-terminus (Suzuki et al., 2009). Wide neuropathological alterations have been described in the postnatal cerebral cortex of *lde/lde* rats, including severe hypomyelination (with a reduced number of mature oligodendrocytes) and a significant reduction in cell populations of astrocytes and microglia (Tochigi et al., 2019). In the present study, histological and immunohistological analyses of developing cerebral cortex of *lde/lde* rats investigated at perinatal age revealed a defective migration of immature neuron and delayed formation of cortical layers, overlapping the identified human pathological features. In conclusion, our study indicates that loss of Wwox could alter a number of transcriptionally regulated genes implicated in cytoskeleton organization and microtubule assembly, leading to cellular and sub-cellular defects of cerebral cortication. These findings highlight a conserved role of *WWOX* in regulating neuronal migration and CNS development across different species. Experimental studies using WOREE individuals-derived forebrain organoids, which can model critical human fetal developmental processes, could help to understand the various phases of cortical development regulated by *WWOX*, and to assess the different molecular pathways implicated in the pathological features of this severe brain developmental disorder.

## Data Availability Statement

The datasets generated for this study can be found in the GEO database (GSE126075) and the ClinVar database (VCV000241105).

## Ethics Statement

The studies involving human participants were reviewed and approved by “Giannina Gaslini” Children’s Hospital Institutional Review Board. Written informed consent to participate in this study was provided by the participants’ legal guardian/next of kin. The animal study was reviewed and approved by Animal Care and Use Committee of Nippon Veterinary and Life Science University. Written informed consent was obtained from the individual(s), and minor(s)’ legal guardian/next of kin, for the publication of any potentially identifiable images or data included in this article.

## Author Contributions

MI: acquisition and interpretation of genetic data, and drafting of the manuscript. AA and SB: immunohistochemistry, immunofluorescence, interpretation data, and drafting of the manuscript. YT: animal model studies, interpretation of data, and drafting of the manuscript. KK and AB: transcriptomic analyses. FB and EF: histopathological studies. AT and VN: NGS analysis. MSe and DP: brain magnetic resonance imaging. LM, AR, and MSc: clinical phenotyping, assessment, and follow-up. GB: sanger sequencing validation and segregation studies. PS and CM: critical revision of manuscript and obtaining funding. FZ: interpretation of genetic data, reviewing of the manuscript, critical revision of manuscript, and obtaining funding. HS: supervision of animal model studies and reviewing of the manuscript. VS: design of the study, final acquisition, interpretation of clinical data, and drafting and reviewing the manuscript. All authors contributed to the manuscript and approved the submitted version.

## Conflict of Interest

The authors declare that the research was conducted in the absence of any commercial or financial relationships that could be construed as a potential conflict of interest.

## Footnotes

1 www.homozygositymapper.org

2 https://www.gsea-msigdb.org/gsea/msigdb
