# Supplementary material for: Loss of Wwox Perturbs Neuronal Migration and Impairs Early Cortical Development
Source: Front Neurosci. 2020 Jun 11;14:644. doi: 10.3389/fnins.2020.00644 (PMC7300205; doi:10.3389/fnins.2020.00644)
Supplement: Supplementary file 1 [file Data_Sheet_1.pdf]

Supplementary Section

Supplementary Material

Immunostaining of WWOX-deficient foetal brain

Tissue blocks of brain parietal coronal sections of the WWOX-deficient foetus terminated at the 21th gestational week and the gestational age-matched male control were dewaxed (2 washes in xylol for 10 min, 2 washes in ethanol 100% for 5min, 5 min in ethanol 95%, 5min in ethanol 90%, 5 min in ethanol 70%) and then rehydrated in distilled water for 5 minutes. The antigen unmasking was performed with citrate solution (10nM sodium citrate buffer, pH 6.0) maintaining at just below boiling temperature for 10 minutes. After washing in distillated water, sections were incubated at room temperature (RT) for 10 minutes with 3% hydrogen peroxide to quench endogenous peroxidase activity. After washing, sections were blocked for 1 hour with 5% Foetal BovinSerum (FBS) in 1X Tris Buffered Saline (TBS) /0.05%Tween 20 (blocking solution), and then incubated with primary anti-TUBA1A antibodies (HPA043684, Sigma-Aldrich, USA) 1:50 overnight at 4°C in a humidified chamber. Sections were washed three times with 0.05% Tween20/1X TBS, incubated with the secondary antibody Alexa Fluor 488-coniugated goat anti-rabbit (Thermo Fisher Scientific, Monza, Italy) diluted 1 hour in blocking solution at RT, washed and mounted with DAPI Fluoromount-G (SouthernBiotech, USA & Canada). Images were taken at 40Xmagnification with AxioVision SE64 Rel.4.9.1 (Carl Zeiss Microscopt GmbH, Jena, Germany). Three region of interest (ROI) were analysed for fluorescence quantification with ImageJ software. The ROIs were positioned in the different part of cortex: molecular layer (ML), external granular layer (EGL).

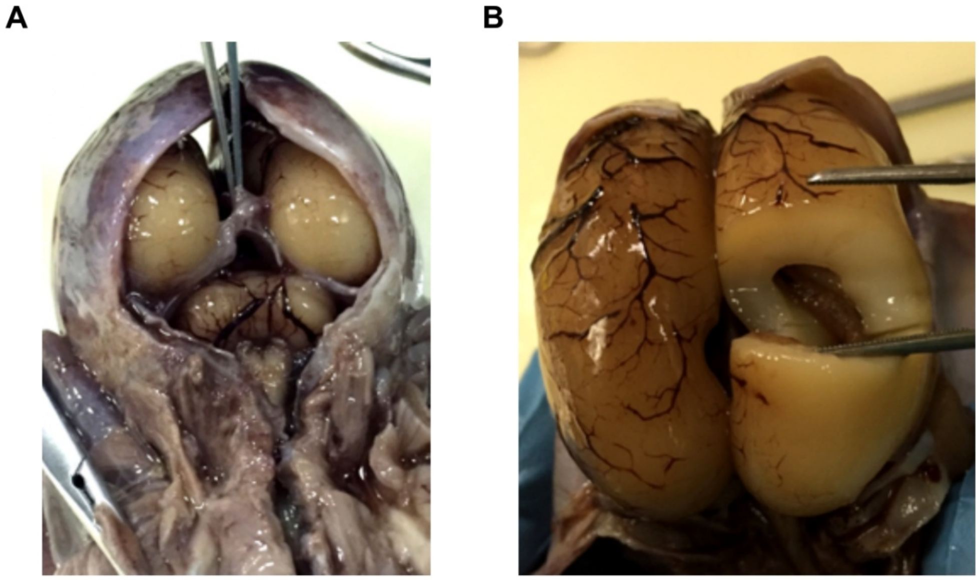

Supplementary Figure S1. Post-mortem image of foetal brain at 21th gestational age.

(A)Image showing evidence of cerebellar vermis hypoplasia (B) No macroscopic abnormalities or malformations were detected in the brain of the WOREE affected foetus.

A

Kinesins

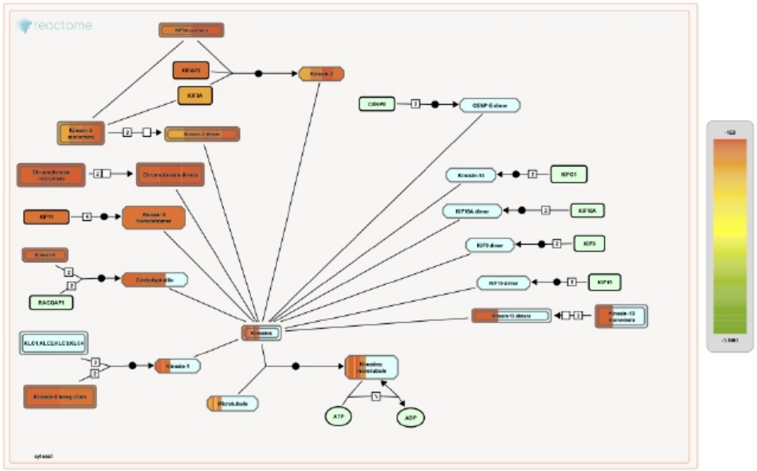

B

Entities found in the analysis (29)

Input	UniProt Id	logFC ne...
ITFG1	Q9Y496	-1.82e+00
KIF21B	O75037	-1.10e+00
KIF1B	O60333	-1.84e+00
KIF3C	O14782	-1.03e+00
KIF21A	Q7Z4S6	-1.63e+00
KIF1A	Q12756	-2.78e+00
KIF3B	O15066	-2.18e+00
KIF5C	Q6PKD1	-1.32e+00
KIF3A	Q9Y496	-3.59e+00
KIF5B	P33176, Q66K46	-1.80e+00
KIF5A	Q12840	-1.41e+00
KIFAP3	Q92845	-1.77e+00
TUBB6	Q9BUF5	-2.36e+00
KIF23	Q02241	-1.58e+00
KIF22	Q14807	-1.32e+00
TUBB4B	P04350, P68371	-1.96e+00
TUBB2A	Q13885, Q9BVA1	-1.84e+00
TUBB2B	Q13885, Q9BVA1	-1.72e+00
KIF20A	O95235	-1.45e+00
KIF2C	Q8N4N8, Q99661	-1.96e+00
KIF16B	Q96L93	-1.19e+00
KIF2A	O00139, Q8N4N8, Q99661	-1.29e+00
KIF4B	O95239, Q2VIQ3	-1.13e+00
KIF4A	O95239	-1.02e+00
KIF20B	Q96Q89	-1.03e+00
TUBB3	Q13509	-3.96e+00
KIF11	P52732	-1.85e+00
TUBA1A	P68363, Q71U36	-3.53e+00
TUBA1B	P68363	-3.36e+00

Supplementary Figure S2. Neural migration-related genes found in the Reactome database (A, B)

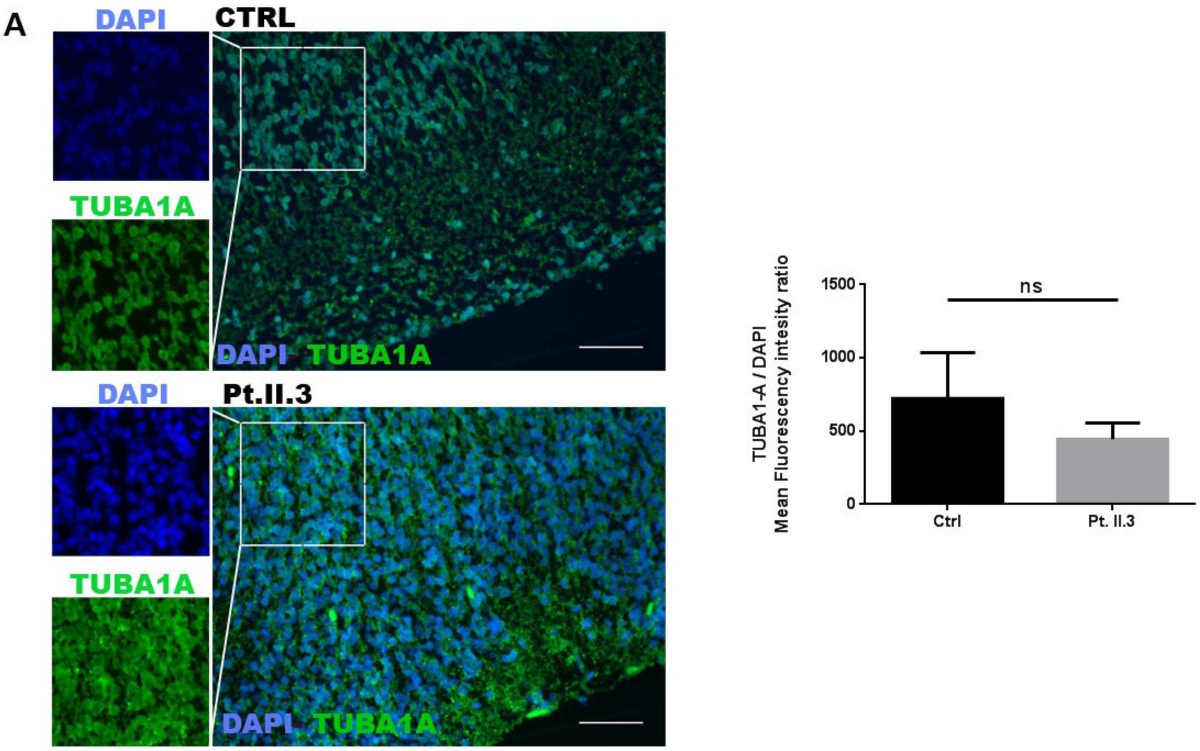

Supplementary Figure S3. Immunostaining of WWOX-deficient foetal brain

(A) Immunofluorescence analysis of human cortex at 21th gestational week. Tuba1a was detected by Alexa 488 (green) conjugated secondary antibody (scale bars 50 µm). In the upper panel a control of 21th gestational week and in the (lower panel) Individual (Pt.) II.3. In the *WWOX*-deficient developing brain a disorganization of cortical layers compared with control is noticed and abnormal distribution of DAPI (blue) staining is noticed. Graph of quantification of mean fluorescence intensity of Tuba1a normalized on the number of nuclei displayed a slight reduction of intensity in individual (Pt.) II.3 compared to the control. N= 3 ROIs were analyzed for each genotype. Data were represented ad mean ± standard error of the mean (SEM.) $p= 0.5714$ (ns $\geq$ 0.05), Mann Whitney's U-test.
